# Another step of the APXPS evolution taken at MAX IV synchrotron

**DOI:** 10.1107/S1600577521002095

**Published:** 2021-02-25

**Authors:** Slavomir Nemšák

**Affiliations:** a Advanced Light Source, Lawrence Berkeley National Laboratory, One Cyclotron Road, Berkeley, CA 94720-8196, USA

**Keywords:** APXPS, *operando*, *in situ*, synchrotron, catalysis, IR, beamline

## Abstract

The article by Zhu *et al.* [*J. Synchrotron Rad.* (2021), **28**, 624–636] published in this journal issue presents the setup and specifications of the new soft X-ray beamline HIPPIE, dedicated to ambient-pressure X-ray photoelectron spectroscopy at the MAX IV Laboratory.

In the past several decades, X-ray photoelectron spectroscopy (XPS) has been established as a versatile tool to investigate chemical and electronic properties of materials. Its principle is based on a photoelectric effect, which was discovered by Hertz (1887 ▸) and explained later by Einstein (1905 ▸). However, a practical technical implementation of the technique had to wait until the 1950s, when Siegbahn *et al.* carried out their first pioneering experiments (Siegbahn & Svartholm, 1946 ▸).

XPS uses X-ray photon beams, which excite photoelectrons that get emitted from a material. Due to a strong interaction of electrons with solids, liquids, and also gases, the technique was initially designed to work with solid samples in ultra-high vacuum only. Even though the early experiments on liquids performed by Siegbahn *et al.* were carried out in the 1970s (Siegbahn & Siegbahn, 1973 ▸), it was the development of modern differentially pumped hemispherical analyzers with focusing pre-lens (Figure 1 ▸) in the early 2000s that paved the way to widespread deployment of XPS systems capable of measuring in ambient pressures (typically tens of mbar) around the world (Salmeron & Schlögl, 2008 ▸).

In the paper by Zhu *et al.* (2021 ▸), a new ambient pressure XPS (APXPS) setup installed at the HIPPIE beamline in MAX IV laboratory in Lund, Sweden, is introduced. It is a state-of-the-art system that was designed to be both modern and versatile to serve a wide scientific community. HIPPIE is an undulator beamline, which covers energies ranging from 250 to 2200 eV and provides high flux (>10^12^ photons s^−1^) even at high energy resolving powers (∼10000). The upper boundary of this energy range is especially important due to the fact that it is the higher kinetic energy of photoelectrons that is needed to overcome attenuation of the electron signal in the gas or liquid environments. Simply speaking, the ability to perform XPS experiments at higher pressures relies on using higher photon excitation energies, pushing the boundaries from soft to hard X-ray excitation.

In order to cover a variety of scientific disciplines, a modular setup allows switching between two concepts: a cell-in-cell design and a backfill ambient pressure chamber. The first one focuses on solid/gas interface studies, catalysis, and rapid gas reactions analysis. The second one specializes in liquid chemistry and *operando* electrochemistry.

Another highlight of the instrument is the multimodal approach that includes infrared reflection absorption spectroscopy (IRRAS), which had been demonstrated earlier (Head *et al.*, 2017 ▸), but at HIPPIE is now an integral part of the experiment. The combination of the high photon flux of the HIPPIE beamline and the fast collection of the electron signal using a CMOS camera enables high-rate spectra acquisition in ∼ms time-scales. Apart from static solid and liquid samples, the setup can also make use of a microjet liquid delivery. Last, but not least, the gas handling system provides automatic mixing of up to eight gases in the pressures ranging from ultra-high vacuum to 30 mbar. A small volume of the cell-in-cell design allows a rapid gas exchange in a matter of seconds.

The authors demonstrate the capabilities of the newly built instrument using several scientific examples. The first one is monitoring hydrogen gas intercalation under graphene flakes in real time. These time-resolved experiments show how the gas delivery system providing a train of pressure pulses of hydrogen and oxygen gases works together with the high spectra acquisition rate. Spectral signatures of graphene with and without intercalated hydrogen are successfully time-correlated with the arrival of hydrogen gas pulses.

Another demonstration uses a lithium/lithium cobalt oxide battery in an *in situ* electrochemical cell. The lithium cobalt oxide electrode was monitored during charging/discharging of the battery. Thanks to the chemical specificity of the APXPS, the team of authors observed a change in the oxidation state as well as electronic structure of the cobalt present in the electrode as Li was extracted/incorporated into the electrode.

The last scientific example presented in the paper is relevant to catalytic studies. Adsorption of carbon monoxide on platinum single crystal is studied both by APXPS and IRRAS. Combination of these two methods permitted unambiguous identification of carbon monoxide occupying bridge and top adsorption sites of Pt (111) and their population under changing carbon monoxide background pressure.

These three scientific demonstrations showcase the versatility and the strengths of this newly built experimental setup at MAX IV synchrotron. The field of APXPS has undergone dramatic developments in the last two decades. Faster acquisitions, a use of multiple complementary probes and a high level of the automation makes the HIPPIE facility another successful iteration in this highly evolving field and a significant achievement, which will benefit all prospective users and the scientific community as a whole.

## Figures and Tables

**Figure 1:**
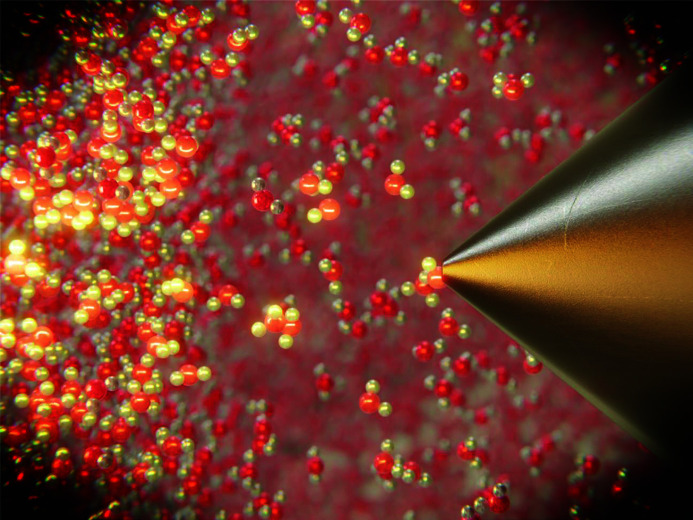
An artistic view of the ambient pressure hemispherical analyzer entrance aperture in a gaseous and liquid environment. Credit: Tomas Duchon, https://micronano.net/.
